# A 2-Benzylmalonate Derivative as STAT3 Inhibitor Suppresses Tumor Growth in Hepatocellular Carcinoma by Upregulating β-TrCP E3 Ubiquitin Ligase

**DOI:** 10.3390/ijms22073354

**Published:** 2021-03-25

**Authors:** Ting Peng, Orawan Wonganan, Zhonghui Zhang, Jialing Yu, Ruiying Xi, Yu Cao, Apichart Suksamrarn, Guolin Zhang, Fei Wang

**Affiliations:** 1 Center for Natural Products Research, Chengdu Institute of Biology, Chinese Academy of Sciences, Chengdu 610041, China; pengting@cib.ac.cn (T.P.); orawan.wog@mahidol.ac.th (O.W.); yujl@cib.ac.cn (J.Y.); xiruiying18@mails.ucas.ac.cn (R.X.); caoyu@cib.ac.cn (Y.C.); 2 University of Chinese Academy of Sciences, Beijing 100049, China; 3 School of Chemistry Engineering, Sichuan University, Chengdu 610041, China; 2018223075220@stu.scu.edu.cn; 4 Department of Chemistry and Center of Excellence for Innovation in Chemistry, Faculty of Science, Ramkhamhaeng University, Bangkok 10240, Thailand; s_apichart@ru.ac.th; 5 Xiongan Institute of Innovation, Chinese Academy of Sciences, Hebei 071700, China

**Keywords:** hepatocellular carcinoma, signal transducer and activator of transcription 3, β-catenin, β-transducin-containing repeat protein, nuclear factor kappa-B, 2-benzylmalonate

## Abstract

The aberrant activation of a signal transducer and activator of transcription 3 (STAT3) restrains type I interferon (IFN) α/β-induced antiviral responses and is associated with the development of cancer. Designing specific STAT3 inhibitors will thus provide new options for use as IFN therapy. Herein, we identified a novel small molecule, dimethyl 2-(4-(2-(methyl(phenyl(p-tolyl)methyl)amino)ethoxy)benzyl)malonate (CIB-6), which can inhibit the IFN-α-induced interferon stimulated response element (ISRE) luciferase reporter (IC_50_ value = 6.4 μM) and potentiate the antiproliferative effect of IFN-α in human hepatocellular carcinoma (HCC) cells. CIB-6 was found to bind to the STAT3 Src homology 2 (SH2) domain, thereby selectively inhibiting STAT3 phosphorylation without affecting Janus kinases and STAT1/2. CIB-6 also inhibited the migration and invasion of HCC cells by inhibiting the epithelial–mesenchymal transition (EMT) process. Mechanistically, CIB-6 reduced the expression of β-catenin (an EMT key protein) via upregulating β-transducin repeat-containing protein (β-TrCP) and curbed nuclear factor kappa-B (NF-κB) activation through restricting the phosphorylation of the inhibitor of NF-κB (IκB) kinase (IKK) via STAT3 inhibition. Treatment with CIB-6 significantly retarded tumor growth in nude mice with SK-HEP-1 xenografts. In addition, clinical sample analysis revealed that lower β-TrCP and higher β-catenin expression could affect the median survival time of HCC patients. Our findings suggest that CIB-6 could be a new therapeutic strategy for HCC therapy through STAT3-mediated β-TrCP/β-catenin/NF-κB axis.

## 1. Introduction

Viral hepatitis is considered to be an independent factor in the development of hepatocellular carcinoma (HCC); integration of an oncogenic viral genome into the host genome subsequently activates oncogenes and represses tumor suppressor genes, ultimately leading to persistent active hepatitis and hepatic fibrosis [1,2]. Interferons (IFNs) are a family of cytokines with antiviral and immunomodulatory effects that are synthesized by a variety of cells [3]. IFN-α has been employed for many years as a major therapeutic strategy for the treatment of hepatitis B virus (HBV) or hepatitis C virus (HCV), to increase the immune response against cancer in clinical practice [4]. However, IFNs have been replaced or relegated to a second-line therapy due to drug resistance, as it overpowers the curative effect of IFN-α in HCC or induces severe liver decompensation when administered to patients with cirrhosis [5]. The failure of IFN therapy against HCV in patients was found to correlate with an elevated IFN-stimulated gene (ISG) signature [6], suggesting that an aberrant activation of the Janus kinase/signal transducer and activator of transcription (JAK/STAT) pathway exists. In the IFN-α-activated STAT family, STAT1 and STAT2 are well-established mediators of antiviral response. STAT1 is also a known tumor suppressor [7], whereas STAT3 is a well-known oncogene that is involved in the proliferation and survival of cancer cells [8,9]. Therefore, inhibiting STAT3 while maintaining the activation of STAT1/2 may be an acceptable therapeutic approach to enhance the antitumor effects of IFN-α.

The overactivation of STAT3 is associated with decreased survival and a high risk of recurrence of HCC and many other types of cancer [10,11]. Constitutive activation of STAT3 initiates transcription of a range of downstream oncogenes, such as β-catenin (encoded by *CTNNB1*) in the Wnt pathway and nuclear factor-κB (NF-κB), frequently hyperactivated in cancer to promote tumor growth or pro-oncogenic inflammation [12,13]. Therefore, selectively disrupting or blocking the intracellular signaling of oncogenes that are triggered in the STAT3-activated context may promote progress in the development of cancer treatments. β-transducin repeat-containing protein (β-TrCP, encoded by *BTRCP*) is a substrate recognition subunit of the SKP1-cullin-1-F-box-protein (SCF)^β-TrCP^ E3 ligase that plays an important role in cell division and various signaling pathways essential for tumorigenesis [14]. β-TrCP recognizes the phosphorylated motif in many specific protein substrates, such as β-catenin and inhibitor of NF-κB (IκB) proteins. Despite its regard as an oncoprotein, the role of β-TrCP in tumorigenesis is cell- and tissue-dependent [15]. However, the upstream signaling pathways governing the activation or inactivation of β-TrCP and SCF^β-TrCP^ E3 ligase remain unclear. Blocking JAK2 activity could upregulate mRNA and protein expression levels or the activity of β-TrCP [16]. However, the role of STATs in this process is still unclear.

Given the important role of STAT3 induced by IFNs in antitumor and immunomodulation, several STAT3 inhibitors have been discovered. However, many exhibit suboptimum potency or pharmacokinetic parameters. For example, the peptidomimetics of STAT3 exhibited relatively less druggable properties, which have resulted in a shift to the discovery and development of small molecules. The importance of the Src homology 2 (SH2) domain in STAT3 dimerization and DNA binding has led to the discovery of STAT3 inhibitors for SH2 domain interactions. However, all STAT proteins contain an SH2 domain (residues Try580 to Glu680), a 100 amino acid sequence containing three solvent-accessible sub-pockets that can be targeted by small-molecule inhibitors [17,18]; the SH2 domain sequence homology of STAT3 and STAT1 is 78% [19]. The potential for cross-reacting and simultaneous suppression of STAT1 and STAT3 activity by one inhibitor is high [9]. Thus, inhibiting STAT3 to block tumor growth without affecting STAT1 is a challenging task. To selectively inactivate STAT3, we employed a cell screening model to identify the small molecule, CIB-6, which specifically inhibits STAT3 activation without affecting STAT1/2 and highlight the potential of using IFN in a combination anti-cancer therapy.

## 2. Results

### 2.1. CIB-6 Inhibits the Interferon Stimulated Response Element (ISRE) Signal Induced by IFN-α without Upstream Kinase and STAT1/STAT2 Inhibition

**Figure 1:**
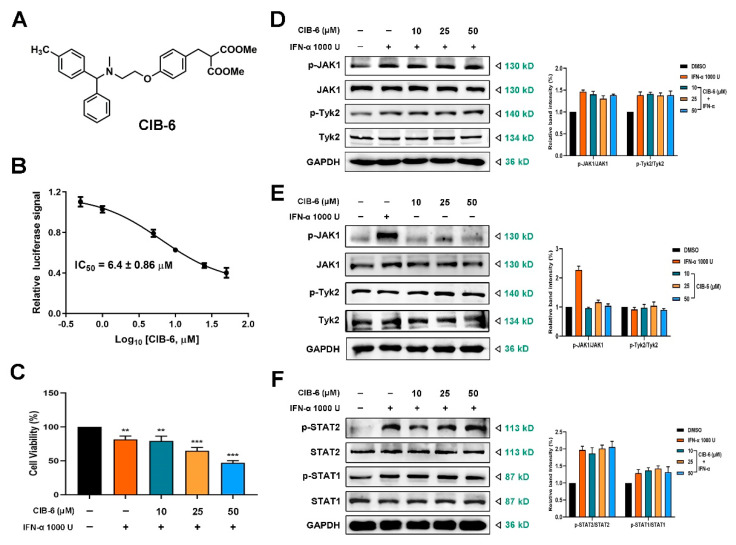
CIB-6 inhibits the interferon stimulated response element (ISRE) signal induced by interferon (IFN)-α without upstream kinase and signal transducer and activator of transcription 1 (STAT1)/STAT2 inhibition. (**A**) The chemical structure of CIB-6. (**B**) HepG2-ISRE-luc2 cells were seeded in 96-well plates, treated with different concentration of CIB-6 for 24 h, and administered 1000 U/mL IFN-α. The IC_50_ value of CIB-6 was calculated. Results are representative of three experiments. (**C**) HepG2 cells were treated with the indicated concentrations of CIB-6 and IFN-α for 24 h. The data are presented as mean ± SD of three independent experiments. (** *p* < 0.01 and *** *p* < 0.001 as compared with DMSO). The SK-HEP-1 cells were treated with the indicated concentrations of CIB-6 for 24 h. (**D**,**E**) The cell lysates were immunoblotted with antibodies against phosphorylated and total proteins of Janus kinase 1 (JAK1) and Tyk2 with or without 1000 U/mL IFN-α stimulation. (**F**) The cell lysates were immunoblotted with antibodies against phosphorylated and total proteins of STAT1 and STAT2.

During the screening of 1431 natural products and synthesized analogues on type I IFN stimulation by using HepG2-ISRE-luc2 cells [20], we found a group of analogs have effects on IFN-α-induced-ISRE luciferase reporter expression. The structures of 15 analogs are presented in Figure S1A. Based on the results, compounds **6**, **12**, **14**, and **15** were identified to decrease ISRE luciferase reporter expression induced by IFN-α (Figure S1B). After comprehensive consideration of the synthesis yield and inhibitory effect of the compounds, compound 6 (dimethyl 2-(4-(2-(methyl(phenyl(p-tolyl)methylamino)ethoxy)benzyl)malonate) CIB-6) (Figure 1A) was selected for further activity testing. CIB-6 significantly inhibited the expression of the ISRE reporter in a concentration-dependent manner, with an IC_50_ value of 6.4 ± 0.86 µM (Figure 1B). By assessing the effect of CIB-6 on IFN-α-induced suppression of cell proliferation, we found that CIB-6 combined with IFN-α significantly inhibited HepG2 cell viability in a concentration-dependent manner (Figure 1C).

Canonical IFN-α signaling activates the JAK/STAT pathway and SK-HEP-1 cells are known to have higher levels of phospho-STATs than HepG2 cells [21]. Therefore, we sought to determine the effect of CIB-6 on the phosphorylation of JAK1 and Tyk2. CIB-6 had no effect on the phosphorylation of JAK1 and Tyk2 after with or without IFN-α stimulation in SK-HEP-1 cells (Figure 1D,E), excluding the possibility that CIB-6 acts as an inhibitor of JAKs. Further, this compound did not affect the phosphorylation of STAT1 and STAT2 stimulated by IFN-α (Figure 1F).

### 2.2. CIB-6 Inhibits IFN-α Induced STAT3 Signaling

**Figure 2:**
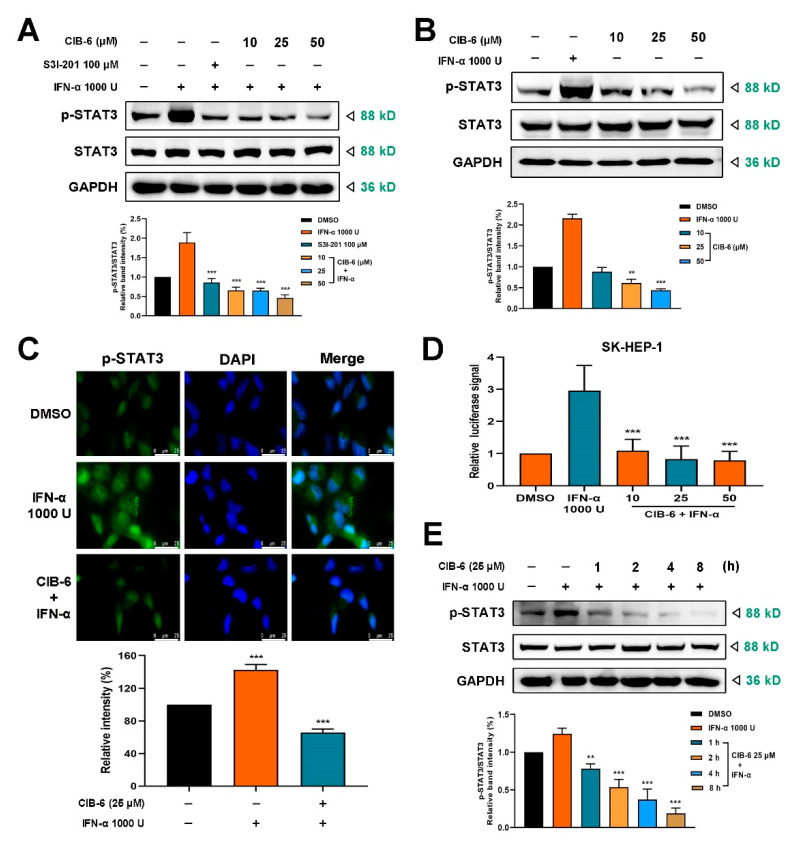
CIB-6 inhibits IFN-α induced STAT3 phosphorylation. The SK-HEP-1 cells were treated with the indicated concentrations of CIB-6 for 24 h and 1000 U/mL IFN-α for 0.5 h. (**A**) The cell lysates were immunoblotted with antibodies against phosphorylated and total proteins of STAT3. The STAT3 inhibitor, S3I-201 (100 µM, treat for 24 h), was employed as the positive control. Bar graph shows quantified levels of p-STAT3/STAT3 from three independent experiments (*** *p* < 0.001 as compared with IFN-α). (**B**) The cell lysates were immunoblotted with antibodies against phosphorylated and total proteins of STAT3 after treated with the indicated concentrations of CIB-6 for 24 h alone. Bar graph shows quantified levels of p-STAT3/STAT3 from three independent experiments (** *p* < 0.01 and *** *p* < 0.001 as compared with DMSO). (**C**) The SK-HEP-1 cells were fixed and analyzed via an immunofluorescence assay (×100). The fluorescence intensity was quantified using ImageJ; data are presented as mean ± SD of triplicate experiments. *** *p* < 0.001 indicates significant differences relative to the vehicle-treated control group. (**D**) After SK-HEP-1 cells were transfected with the pGL4.26-3×STAT3 plasmid, luciferase repression of CIB-6 treated cells compared to the control or IFN-α was calculated and plotted as a fold change in luciferase activity. The luciferase of the empty vehicle was used to normalize the luciferase values. The data are presented as mean ± SD of three independent experiments (*** *p* < 0.001 as compared with IFN-α). (**E**) The SK-HEP-1 cells were treated with 25 µM CIB-6 for the indicated period. Thereafter, immunoblotting was performed to detect the phosphorylated STAT3 expression and total proteins of STAT3 after IFN-α induction. GAPDH was used as an internal control. Bar graph shows quantified levels of p-STAT3/STAT3 from three independent experiments (** *p* < 0.01 and *** *p* < 0.001 as compared with IFN-α).

We proceeded to determine the effect of CIB-6 on STAT3. Compared with IFN-α treatment alone, this compound significantly decreased the tyrosine phosphorylation of STAT3 induced by IFN-α, and the phosphorylation inhibitory effect of 10 μM CIB-6 on STAT3 was comparable with that of 100 μM S3I-201, a STAT3 inhibitor (Figure 2A). To further verify the effect of CIB-6 on STAT3 signal transduction, SK-HEP-1 cells were treated with CIB-6 alone without IFN-α stimulation. As shown in Figure 2B, CIB-6 strongly inhibited STAT3 phosphorylation in a concentration-dependent manner.

In addition, the inhibition of STAT3 nuclear translocation by CIB-6 was examined using immunofluorescence staining. Based on the results, phosphorylated STAT3 in the nucleus was reduced following treatment with CIB-6 (Figure 2C). Based on the previous experimental results of reduced STAT3 phosphorylation, we hypothesized that phosphorylated STAT3 nuclear translocation was reduced. To further investigate the effect of CIB-6 on STAT3 transcription, we transiently transfected the pGL4.26-3×STAT3 plasmid or empty vehicle (Scheme of plasmids illustrated in Figure S2A) into liver cancer cells. The luciferase signal in the pGL4.26-3×STAT3 plasmid apparently increased transactivation after IFN-α treatment; however, the reporter activity was markedly inhibited by CIB-6 in a concentration-dependent manner in SK-HEP-1 cells (Figure 2D) and HepG2 cells (Figure S2B). Furthermore, the western blot results revealed that CIB-6 inhibited STAT3 phosphorylation in a time-dependent manner and inhibited the stimulation of STAT3 by IFN-α from 4 h (Figure 2E). As CIB-6 inhibited the activation of STAT3, we further determined its effect on IL-6-induced STAT3 signaling. As shown in Figure S2C, CIB-6 could impede the activation of STAT3, which aligns with the results of IFN-α stimulation.

### 2.3. CIB-6 Directly Interacts with STAT3

**Figure 3:**
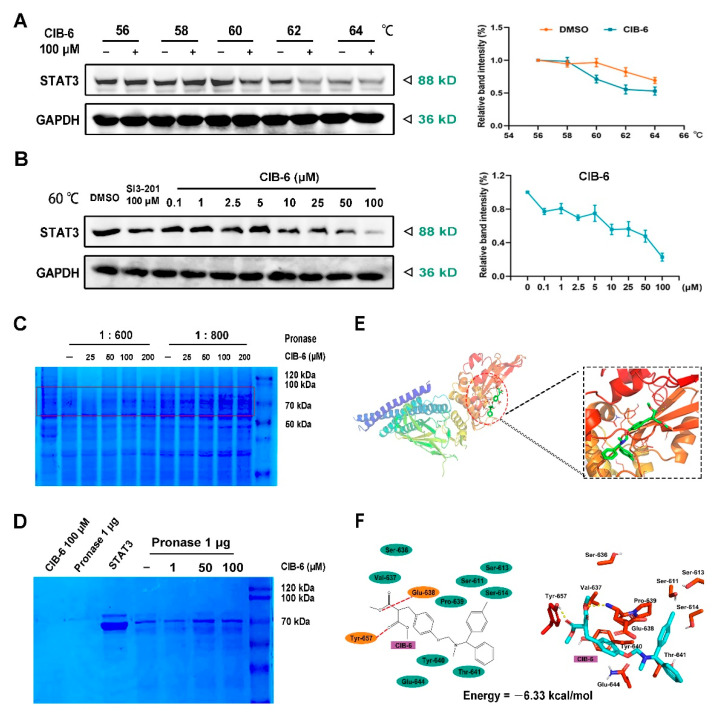
CIB-6 directly interacts with STAT3. The stabilization effect of CIB-6 on STAT3 in SK-HEP-1 lysates was evaluated by Cell Thermal Shift Assay (CETSA) at different temperatures (**A**) and different concentrations (**B**). GAPDH was employed as the loading control. The ordinate in the quantification line chart is the percentage of the relative gray value. Graphic data were run in triplicate and are presented as mean ± SD. SDS-PAGE Coomassie-staining confirmed the stabilization of STAT3 by CIB-6 in SK-HEP-1 cell lysates (pronase: STAT3 1:600 or 1:800) (**C**) as well as the stabilization of pure recombinant expressed STAT3 protein (pET15b-STAT3 (Δ127-722)) by CIB-6 (pronase: STAT3 1:800) (**D**) after pronase digestion in the Drug Affinity-Responsive Target Stability (DARTS) assay. (**E**) Overview of the binding mode of CIB-6 (green) at the STAT3 crystal structure (Protein Data Bank (PDB) ID: 6NJS). (**F**) Two-dimensional (2D) interaction diagram shows all amino acids in the STAT3 SH2 domain active site involved in the formation of H-bonds and hydrophobic interactions with CIB-6.

We further sought to determine whether CIB-6 interacts with STAT3 in cells via a Cell Thermal Shift Assay (CETSA) assay; this assay is based on the biophysical principle of ligand-induced thermal stabilization of target proteins. CIB-6 stimulated STAT3 protein instability at 60 °C (Figure 3A). Additionally, STAT3 levels were found to decrease with increasing CIB-6 concentration at 60 °C (Figure 3B). In the Drug Affinity-Responsive Target Stability (DARTS) assay, when cell lysate samples were pretreated with CIB-6 (before different concentrations of pronase treatment), one Coomassie (SimplyBlue)-staining band between the 50- and 100-kDa markers, which was more intense in the CIB-6-treated lysate post-proteolysis than the vehicle control (Figure 3C), was observed. Accordingly, we sought to detect the interaction between recombinant expressed STAT3 protein and CIB-6. As shown in Figure 3D, CIB-6 dose-dependently increased STAT3 protein accumulation. However, no detectable bands were observed in the pure STAT3 protein samples that underwent pronase digestion without CIB-6 protection. Such findings suggest that CIB-6 binding alone could be sufficient to stabilize the bound STAT3 protein in the protease-resistant state.

To understand the binding interactions between CIB-6 and STAT3, a predictive molecular docking study was carried out using the X-ray crystal structure of the STAT3 protein (Protein Data Bank (PDB) ID: 6NJS). The results showed that CIB-6 was inserted in the SH2 domain of STAT3, and the favorable conformation of CIB-6 in STAT3 active site showed an energy value of −6.33 kcal/mol. The two amino acid residues (Glu-638 and Tyr-657) in the STAT3 SH2 domain form hydrogen bonds with CIB-6. Simultaneously, CIB-6 binds to the amino acid residues Ser-611, Ser-613, Ser-614, Ser-636, Val-637, Pro-639, Tyr-640, Thr-641, and Glu-644 through hydrophobic interactions (Figure 3E,F).

### 2.4. CIB-6 Inhibits the Viability, Migration, and Invasion of HCC Cells

**Figure 4:**
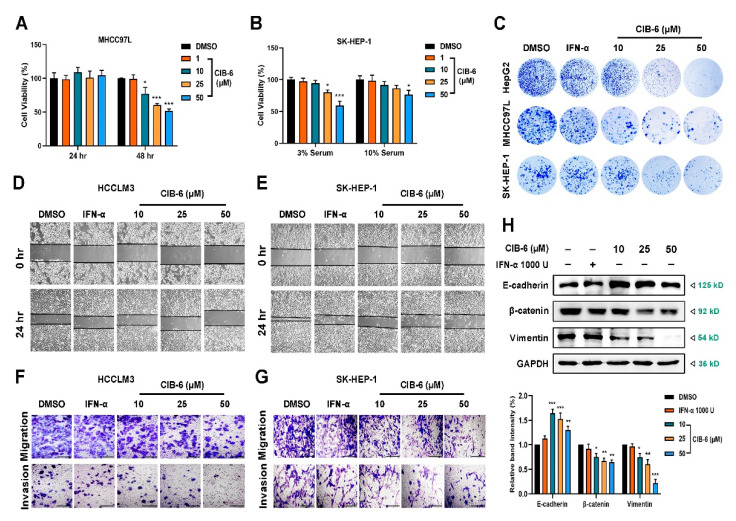
Effects of CIB-6 on the proliferation, migration, and invasion activity of human hepatocellular carcinoma (HCC) cells. (**A**) MHCC97L cell were treated with different concentrations of CIB-6 for 24 h or 48 h under the 10% serum condition (* *p* < 0.05 and *** *p* < 0.001 compared to the DMSO). (**B**) SK-HEP-1 cells were treated with CIB-6 for 24 h under different serum conditions. Cell viability was measured by cell counting kit-8 (CCK-8). All data are presented as mean ± SD of triplicate experiments (* *p* < 0.05 and *** *p* < 0.001 compared to the DMSO). (**C**) CIB-6 (10, 25, and 50 µM) suppressed colony formation in HepG2, MHCC97L, and SK-HEP-1 cells. (**D**,**E**) Monolayers of HCCLM3 and SK-HEP-1 were scratched mechanically and treated with CIB-6 for 24 h (×40). (**F**,**G**) HCCLM3 and SK-HEP-1 cells seeded into the upper chambers (Matrigel embedded for invasion assays) of transwell inserts and treated with CIB-6 for 24 h. Cells were subsequently fixed and stained. Representative images obtained under a light microscope are displayed (×100). (**H**) Effects of CIB-6 on mesenchymal marker (E-cadherin, β-catenin, and Vimentin) expression in SK-HEP-1 cells. Bar graph shows quantified levels of proteins from three independent experiments (* *p* < 0.5, ** *p* < 0.01 and *** *p* < 0.001 as compared with DMSO).

Constitutively-activated STAT3 has been reported to contribute to cancer proliferation and metastasis [22]. Our previous experiments revealed that CIB-6 alone could inhibit STAT3 activation. To confirm the anti-proliferative effects of CIB-6, MHCC97L, and SK-HEP-1 cells were incubated with different concentrations of CIB-6 for different time or in different serum concentration. Based on the results of the cell counting kit-8 (CCK-8), no significant anti-proliferation effect was observed in the MHCC97L cells treated with CIB-6 for 24 h. However, cell viability was remarkably decreased in a dose-dependent manner at 48 h in 10% serum (Figure 4A). Further, only 50 µM CIB-6 inhibited the proliferation of SK-HEP-1 cells (Figure 4B). However, CIB-6 was found to severely restrain the colony formation capacity of HepG2, MHCC97L, and SK-HEP-1. In fact, cells treated with CIB-6 (10, 20, 50 µM) for 7–14 days formed fewer and smaller colonies relative to cells in the control group, whereas the number of colonies in IFN-α was slightly altered (Figure 4C). Such findings suggest that CIB-6 was not cytotoxic to a panel of tumor cells at concentrations that inhibited STAT3 phosphorylation. However, long-term treatment with CIB-6 could inhibit tumor proliferation.

To determine the effects of CIB-6 on the migration and invasion of HCCLM3 and SK-HEP-1, we conducted wound healing and transwell assays. Notably, CIB-6 treatment for 24 h effectively inhibited cell wound closure (Figure 4D,E). Further, the numbers of migrating or invasive cells were significantly reduced by CIB-6 treatment in both cell lines (Figure 4F,G). To further elucidate the molecular mechanism underlying the inhibition of cancer cell invasion and migration caused by CIB-6, we determined the effects of CIB-6 on the expression levels of epithelial–mesenchymal transition (EMT)-associated markers in SK-HEP-1 cells. The expression level of E-cadherin was upregulated while that of β-catenin and Vimentin was downregulated following treatment with CIB-6 (Figure 4H). Collectively, these findings suggest that CIB-6 can suppress cancer cell progression by downregulating EMT in vitro.

### 2.5. CIB-6 Suppresses β-TrCP/β-catenin/NF-κB axis by Inhibiting STAT3

**Figure 5:**
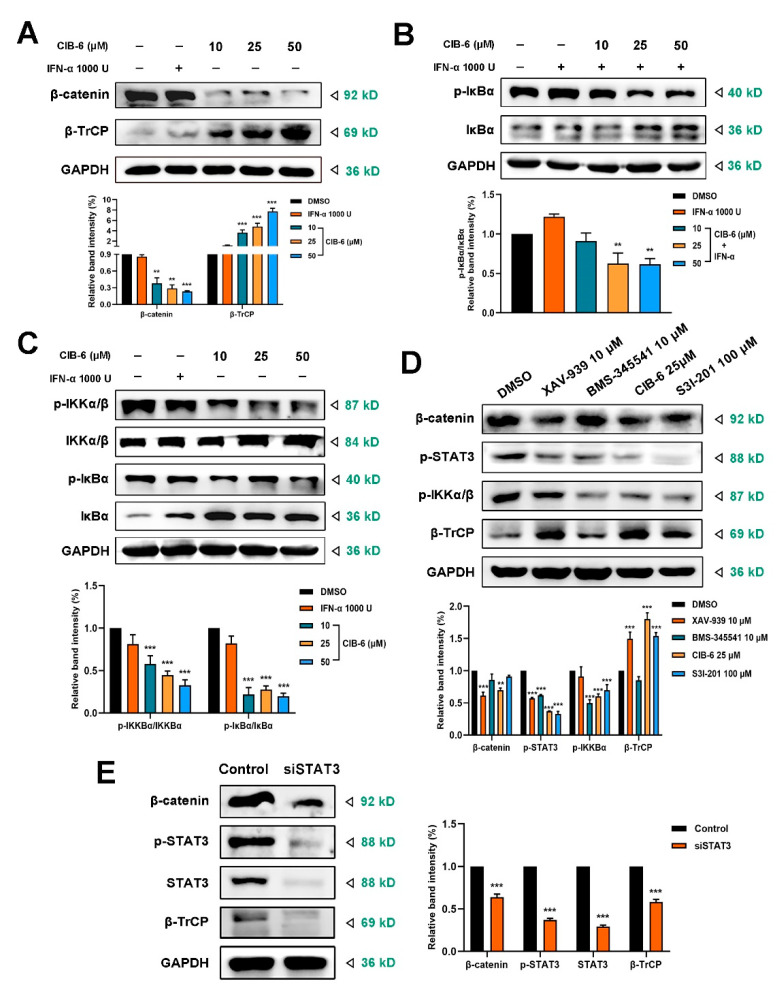
Effects of CIB-6 on the β-transducin repeat-containing protein (β-TrCP)/β-catenin/ nuclear factor kappa-B (NF-κB) axis. (**A**) The SK-HEP-1 cells were treated with the indicated concentrations of CIB-6 for 24 h or stimulated with 1000 U/mL IFN-α for 0.5 h. Cell lysates were immunoblotted with antibodies against β-catenin and β-TrCP. Bar graph shows quantified levels of proteins from three independent experiments (** *p* < 0.01 and *** *p* < 0.001 as compared with DMSO). (**B**,**C**) The SK-HEP-1 cells were treated with the indicated concentrations of CIB-6 for 24 h or stimulated with or without 1000 U/mL IFN-α for 0.5 h. Cell lysates were immunoblotted with antibodies against total and phosphorylated proteins of IκBα and IKKα/β. Bar graph shows quantified levels of p-IKKα/β/IKKα/β and p-IκBα/IκBα from three independent experiments ((**B**), ** *p* < 0.01 as compared with IFN-α; (**C**), *** *p* < 0.001 as compared with DMSO). (**D**) The SK-HEP-1 cells were treated with β-catenin inhibitor (XAV-939, 10 µM, treat for 24 h), NF-κB inhibitor (BMS-345541, 10 µM, and treat for 2 h), CIB-6 (25 µM, treat for 24 h) and STAT3 inhibitor (S3I-201, 100 µM, treat for 24 h). The cell lysates were immunoblotted with antibodies against β-catenin, p-STAT3, p-IKKα/β, and β-TrCP. Bar graph shows quantified levels of proteins from three independent experiments (** *p* < 0.01 and *** *p* < 0.001 as compared with DMSO). (**E**) SK-HEP-1 cells were transiently transfected with siSTAT3. Thereafter, the cell lysates were immunoblotted with antibodies against β-catenin, β-TrCP, STAT3 and phosphorylated STAT3. Bar graph shows quantified levels of proteins from three independent experiments (*** *p* < 0.001 as compared with control).

β-Catenin is a core factor in the EMT process and is activated by phosphorylated STAT3 [23]. However, activated β-catenin can be acceleratively degraded by β-TrCP E3 ligase. It is unclear whether β-TrCP participates in CIB-6-mediated β-catenin signaling. Therefore, we determined the effect of CIB-6 on β-TrCP and its substrate, β-catenin. We found that CIB-6 significantly decreased β-catenin expression but increased β-TrCP expression in the presence or absence of IFN-α stimulation in a concentration-dependent manner (Figure S3A, Figure 5A). CIB-6 also decreased β-catenin expression and increased β-TrCP expression in a time-dependent manner (Figure S3B). IκBα is another substrate of β-TrCP that is degraded by β-TrCP [24]. We examined the effect of CIB-6 on NF-κB signaling that is also associated with STAT3 activation. As shown in Figure 5B,C, CIB-6 significantly inhibited the IκB phosphorylation and stabilized IκB expression in a concentration-dependent manner in the presence or the absence of IFN-α. This excluded the possibility that CIB-6 upregulated β-TrCP to degrade IκB and activate NF-κB pathway. To further determine the effect of CIB-6 on IκB kinase α/β (IKKα/β), we assessed the expression of phosphorylated IKKα/β. The immunoblotting results revealed a significant reduction in phosphorylated IKKα/β after treatment with CIB-6 (Figure 5C). Collectively, these results indicate that CIB-6 could inhibit IKKα/β kinase activity to reduce NF-κB activation.

The mRNA expression levels of STAT3, β-TrCP, and β-catenin were also detected by RT-qPCR. Based on our findings, CIB-6 had no significant effect on STAT3, β-catenin, and β-TrCP transcription (Figure S3C). Such finding indicates that CIB-6 affects β-TrCP and β-catenin expression at the posttranscriptional level. Cells were also treated with the STAT3 inhibitor (S3I-201), β-catenin inhibitor (XAV-939), and NF-κB inhibitor (BMS-345541) to detect their effect on the related proteins. We found that the inhibition of STAT3 and IKKα/β by the NF-κB inhibitor BMS-345541 did not necessarily increase β-TrCP expression (Figure 5D). Furthermore, the β-catenin inhibitor XAV-939 upregulated β-TrCP after β-catenin inhibition. Compared with S3I-201, CIB-6 caused better inhibition of β-catenin and upregulation of β-TrCP. Considering the influence of CIB-6 on β-catenin and β-TrCP after inhibiting STAT3 activity, we transiently transfected with siSTAT3 to detect its effect on these genes. The q-PCR results suggested that the mRNA levels of β-catenin and β-TrCP were not affected after STAT3 silencing (Figure S3D). However, the immunoblotting results showed that both β-TrCP and β-catenin were inhibited when the total STAT3 protein level decreased (Figure 5E). This finding suggests that the inhibition of STAT3 expression differs from that of STAT3 phosphorylation in the regulation of β-TrCP. STAT3 silencing also induced a decrease in the NF-κB pathway signaling (Figure S3E). Collectively, these results indicate that CIB-6 specifically regulates the STAT3-β-TrCP-β-catenin axis and STAT3-NF-κB axis.

### 2.6. CIB-6 Exhibits Antitumor Activity in a Xenograft Mouse Model

**Figure 6:**
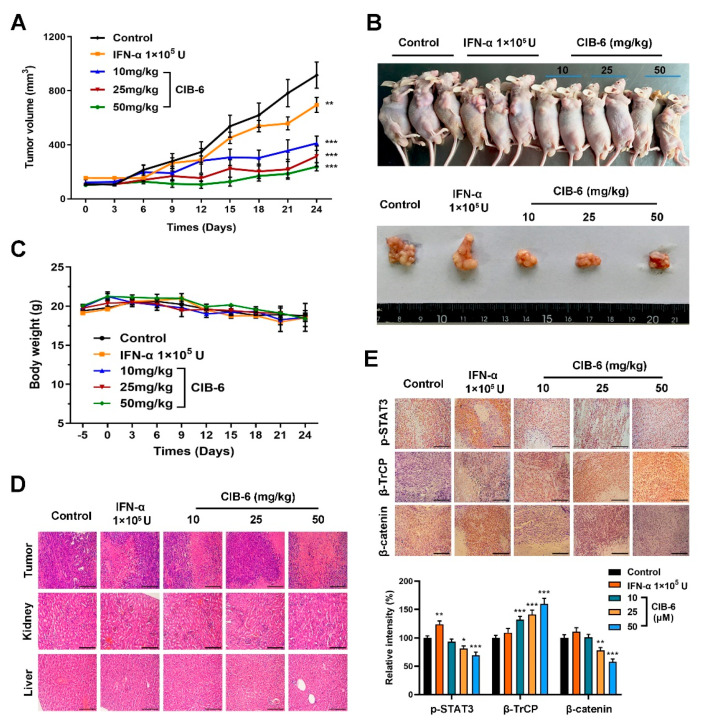
CIB-6 suppresses HCC tumor growth in vivo. (**A**) Tumor volumes were measured every three days with a caliper (*n* = 8, ** *p* < 0.01, *** *p* < 0.001 as compared with control). (**B**) Tumors were excised from mice at the termination of the experiment. Representative images of mice with xenograft tumors were captured with a camera. (**C**) Body weights of mice were measured every three days. (**D**) Hematoxylin–Eosin (H&E) staining of tumor tissues and major organs (liver and kidney). A magnification of ×100 was employed. (**E**) Immunohistochemical analyses of p-STAT3, β-catenin, and β-TrCP expression (×100). The relative intensity of the indicated proteins was semi-quantified using the ImageJ software (* *p* < 0.05, ** *p* < 0.01, *** *p* < 0.001 as compared with control).

To further validate the effects of CIB-6 on HCC cancer progression, xenograft experiments were performed to determine the anti-tumor effects in vivo. The administration of CIB-6 resulted in significantly reduced tumor burdens in the SK-HEP-1 subcutaneous xenograft mice relative to their vehicle-treated and IFN-α-treated counterparts (Figure 6A,B). The body weight of mice also showed a slow and continuous decrease in all groups; however, the change was not statistically significant (Figure 6C). Hematoxylin–Eosin (H&E) staining of tumor tissues and major organs of mice treated with CIB-6 revealed no evident damage (Figure 6D), indicating that there was no systemic toxicity after CIB-6 administration. Immunohistochemistry was subsequently performed to examine paraffin-embedded tumor sections harvested from the mice model. Compared to the vehicle and IFN-α groups, tumor tissues of the CIB-6 group showed decreased levels of p-STAT3 and β-catenin, and the expression level of β-TrCP was increased, thereby aligning with the results observed in vitro. Altogether, these data suggest that CIB-6 is an effective agent for inhibiting HCC tumor growth in vivo.

### 2.7. The Expression Levels of β-TrCP and β-catenin Correlate with the Development of HCC in Humans

**Figure 7:**
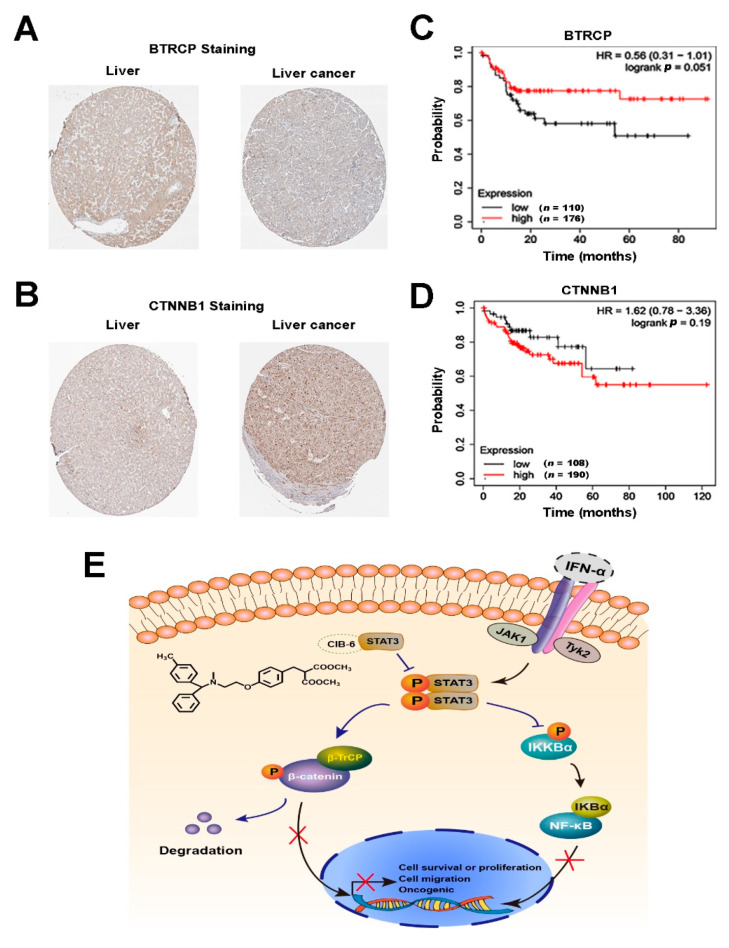
Correlation of β-catenin and β-TrCP expression with human HCC development. (**A**,**B**) Expression of β-TrCP and β-catenin in normal liver tissue and liver cancer tissue. (**C**,**D**) Kaplan–Meier survival curve for β-TrCP and β-catenin in hepatitis-associated HCC patients. (**E**) Schematic summary of the mechanism of CIB-6 in the suppression of HCC. CIB-6 inhibits STAT3 activation to upregulate β-TrCP expression, promote β-catenin degradation, and curb NF-κB activation by restricting IKK phosphorylation, ultimately leading to the suppression of HCC proliferation and metastasis.

To explore the clinical importance of β-TrCP and β-catenin expression in HCC patients, we verified their expression in liver cancer tissues by searching the human protein atlas (https://www.proteinatlas.org/, accessed on 6 October 2020). We found that most HCC patients exhibited lower β-TrCP and higher β-catenin expression. Representative immunohistochemistry (IHC) results are presented in Figure 7A,B. We also performed a clinical analysis to investigate the effect of the abnormal expression of β-TrCP and β-catenin in hepatitis-associated HCC on the patient’s prognosis (https://kmplot.com, accessed on 6 October 2020). The high expression of β-catenin and low expression of β-TrCP in the liver could affect the median survival time of HCC patients; however, the difference was not statistically significant (Figure 7C,D). As a result, a larger sample analysis may be needed to confirm their effect in HCC.

## 3. Discussion

The clinical efficacy of IFN-α in viral hepatitis-associated HCC is often limited by its inability to efficiently induce cell death. However, accumulating evidence suggests that STAT3 is a negative regulator of the IFN-α response and continuously activated STAT3 induces potent cell survival genes that promote inflammation and form a positive feedback to boost tumorigenesis [25]. A recent report revealed that STAT3 is selectively activated by type I IFNs [26], suggesting that viruses may exploit STAT3 to evade IFN-mediated anti-viral immunity and facilitate virus replication [27]. With regards to reduced HCC development, long-term IFN therapy (up to 3.5 years) was not found to provide any benefits to patients [28]. Therefore, inhibiting STAT3 may have significant therapeutic and preventive effects in patients with HCC. In this study, we identified a novel small molecule, CIB-6, that inhibits IFN-α signaling by suppressing STAT3 activation without affecting upstream JAK1 and Tyk2, ultimately ruling out the possibility that CIB-6 acts as a kinase inhibitor to suppress STAT signaling. Previously, CIB-6 was found to exhibit angiotensin-converting enzyme (ACE) inhibitory activity with an IC_50_ value of 3.9 nM; it’s more potent than that of the typical ACE inhibitor (ACEI), captopril (IC_50_ = 7.5 nM) [29]. Interestingly, ACEI drugs have a strong inhibitory effect on tumor angiogenesis and can effectively inhibit tumor growth and metastasis [30,31]. Some ACEIs have been reported to inhibit the NF-κB, Wnt/β-catenin, and STAT3 pathways [32,33], thereby aligning with the findings herein. Whether other ACEIs may also target STAT3 to exhibit their anti-tumor effects should be investigated in a future study.

STAT proteins are thought to be ideal targets in anti-cancer therapy. This is because cancer cells are more dependent on STAT activity than their normal counterparts [34]. However, STAT3 inhibitors must be able to distinguish between STAT3 and STAT1 to be useful in the clinic. The homology between the STAT3 SH2 domain from residues Trp 623 to Tyr 657 and the corresponding residues in STAT1 is proposed to be low. Further, the formation of hydrophobic binding with the Val 637 and Tyr 657 residues is critical for selectivity [35]. Residues Ser 611 and Ser 613, which are quite rigid, also exhibit direct polar interactions with phosphotyrosine 705 (pTyr-705) in STAT3 [36]. In a molecular docking study, CIB-6 forms a hydrophobic bond with residues Ser 611 and Ser 613, implying that the dimerization of STAT3 through reciprocal interaction between the pTyr-705 residue and the SH2 domain may be impaired. No common binding site was found between the STAT1 key residues (Asn 574, Glu 587, Arg 602, Glu 605, Trp 616, Phe 628, Val 631, Tyr 651) [37] in the docking study. Further, the hydrogen bond formed by CIB-6 with residue Val 637 implies the selectivity of CIB-6 for STAT3. Some potent small molecule inhibitors of STAT3, similar to peptidomimetics, form H-bonds with the Ser 611, Ser 613, or Val 637 residue in the binding pocket [38]. For example, S3I-201 formed hydrogen bonds and electrostatic interactions with Ser 611 and Ser 613 as well as a key hydrophobic interaction with Val 637 [39]. Therefore, the binding mode of CIB-6 may be different with S3I-201, which may make CIB-6 more potent than S3I-201 in the inhibition of STAT3. The phosphorylation of Tyr-705 in the SH2 domain is not only necessary for dimerization, but also important for DNA binding. Given the direct binding of CIB-6 to STAT3 in the SH2 domain, we infer that the suppression of Tyr 705 phosphorylation evokes the inhibition of STAT3-DNA interaction; this is consistent with our finding that CIB-6 inhibits STAT3 nuclear translocation and STAT3-responsive reporter expression. The phosphorylation status of STAT3 correlated with the resistance of HCC cells to cetuximab treatment [21], indicating that the downregulation of pSTAT3 by CIB-6 may diminish drug resistance in HCC; however, further investigations are warranted.

β-Catenin nuclear aggregation has been reported in 40–70% of HCC cases [40]; this aggregation is also associated with distant metastasis and chemo-radio-therapeutic resistance in HCC [41]. Activation of STAT3 was previously reported to be involved in the nuclear accumulation of β-catenin; however, the mechanism remains unknown [42]. A canonical approach to accelerate the degradation of β-catenin is mediated by SCF^β-TrCP^ E3 ligases. Endogenous β-TrCP is expressed at low levels and its function can be easily disrupted by the expression of short inhibitory RNA or pseudo-substrates [43]. By comparing databases, we found that patients with a low abundance of β-TrCP have a poor prognosis. Thus, the demand for β-TrCP may exceed its preexisting cellular levels and require a boost to its expression to degrade hyperactive oncogenes in cancer. The degradation of β-catenin may induce a negative feedback regulatory loop to upregulate β-TrCP protein expression at the post-transcriptional level [44]. In this study, we investigated CIB-6, which can induce β-catenin degradation and upregulate β-TrCP protein expression without affecting gene transcription. In addition, the β-catenin inhibitor, XAV-939, was found to induce β-TrCP upregulation. Thus, the inhibition of STAT3 by CIB-6 may stimulate β-catenin degradation, which further induces a negative feedback regulatory loop to upregulate β-TrCP protein expression. Most HCC patients are diagnosed at advanced stages [45]. However, the dysregulation of the Wnt/β-catenin pathway is an early event in hepatocarcinogenesis. Accordingly, CIB-6 can intervene in HCC at an early stage. Strikingly, the β-catenin activated HCC subclass without *CTNNB1* mutation is preferentially associated with chronic HBV infection [46]. Thus, CIB-6 could synergize with IFN-α to treat HBV-related HCC. In addition, the ubiquitin-proteasome system plays an important role in cellular immunity. Therefore, the CIB-6-induced inhibition of STAT3 and increase in E3 ligase β-TrCP expression may be beneficial to enhance the IFN-induced anti-cancer effects in immunotherapy. However, further investigations are warranted.

The use of IFN-α to treat hepatitis-positive HCCs can activate a parallel STAT3-mediated NF-κB signaling pathway [47,48]. The sustained activation of these transcription factors in the same tumor induces a highly overlapping repertoire of proliferative and metastatic gene expression [49] that can enhance metastatic potential by promoting EMT and actuating the inflammation–fibrosis–cancer axis in HCC [50]. Previous research concluded that the inhibition of STAT3 phosphorylation alone is insufficient to inhibit downstream gene expression. As a result, the disruption of multiple transcription factors may be required [51]. IκB is another typical substrate of β-TrCP, which degrades phosphorylated IκB to initiate NF-κB signaling. Interestingly, we found that CIB-6 reduced IκB phosphorylation and caused total IκB protein accumulation, despite increasing β-TrCP expression. According to a recent study, NORE1A forms a complex with β-TrCP, which specifically targets β-catenin, but does not degrade IκB [52]. Therefore, β-catenin degradation may competitively combine with a limited pool of β-TrCP molecules, resulting in insensitivity between IκB and β-TrCP. Other findings have also demonstrated that β-TrCP negatively regulates IKK activation [53], which is consistent with the restrained activation of NF-κB observed when the phosphorylation of IKK is reduced. Both STAT3 and NF-κB participate in the EMT of cancer cells [23,54], which aligns with our findings that CIB-6 suppresses the proliferation of HCC cells in vitro and in vivo, and inhibits EMT-mediated HCC migration. The activation of STAT3 dysregulates the reparative functions of fibroblasts in the human lung. In fact, STAT3 is considered to be a therapeutic target for anti-lung fibrosis [27]. Recently, pulmonary fibrosis was found to be fatal in Corona Virus Disease 2019 (COVID-19) patients. However, early treatment with IFN before viral peak results in a protective effect while late treatment may lead to increased inflammation. As a result, the protective effects of IFN are controversial [55]. Angiotensin-converting enzyme 2 (ACE2) is the main host cell receptor for the entry of syndrome-corona virus 2 (SARS-CoV-2) and is highly expressed in respiratory epithelial cells. Considering the dual inhibitory effect of CIB-6 on STAT3 and ACE, CIB-6 could be further explored for development as a new anti-COVID-19 drug.

In summary, CIB-6 targets the SH2 domain of STAT3 to ultimately inhibit STAT3 phosphorylation and nuclear translocation. Furthermore, the upregulation of β-TrCP and disruption of the oncogenic loops of the STAT3–β-catenin–NF-κB signaling pathway by CIB-6 were found to prevent HCC tumorigenesis in vitro and in vivo (Figure 7E). Such findings not only reveal the function of STAT3 in the regulation of the β-TrCP/β-catenin/NF-κB axis, but also highlight the need to further investigate CIB-6 as a new therapeutic strategy for the treatment of HCC.

## 4. Materials and Methods

### 4.1. Reagents

IFN-α (recombinant human IFN-2α) was purchased from GenScript Bio-Tech Co., Ltd. (Nanjing, China). The specific primary antibodies used in this study as follow: phospho-JAK1 (Tyr 1022, Cat No. 11149), phospho-Tyk2 (Tyr1054, Cat No. 11148), Tyk2 (Cat No. 22852), phospho-STAT2 (Tyr690, Cat No.11536), phospho-STAT3 (Tyr705, Cat No. 11045), BTRC (β-TrCP, Cat No. 32369), and IKKα/β (Cat No. 41057) purchased from Signalway Antibody (College Park, MD, USA); anti-JAK1 (Cat No. 3332S), phospho-IκBα (Ser32/36, Cat No. 9246S) and phospho-IKKα/β (Ser176/180, Cat No. 2697T) purchased from Cell Signaling Technology (Danvers, MA, USA); STAT1 (Cat No. ET1606-39), phospho-STAT1 (Ser727, Cat No. ET1611-20) and STAT3 (Cat No. ET1607-38) purchased from HuaBio-Antibodies (Hangzhou, China); STAT2 (Cat No. 16674-1-AP), β-catenin (Cat. 51067-2-AP), IκBα (Cat. 10268-1-AP), E-cadherin (Cat No. 20874-1-AP), Vimentin (Cat No. 10366-1-AP) and GAPDH (Cat No. 10494-1-AP) purchased from Proteintech (Chicago, IL, USA). STAT3 inhibitor (S3I-201, Cat. S1155), β-catenin inhibitor (XAV-939, Cat. S1180), and NF-κB inhibitor (BMS-345541, Cat. S8044) purchased from Selleck (Houston, TX, USA). CIB-6 is derived from our compound library, which has been reported previously [29].

### 4.2. Cell Culture

All cells were obtained from the Chinese Academy of Sciences Shanghai Cell Bank, and all cells were authenticated via short tandem repeat (STR) profiling. The human HCC cells, MHCC97L (low metastatic potential), HCCLM3 and SK-HEP-1 (high metastatic potential) were cultured in Dulbecco’s Modified Eagle Medium (DMEM, HyClone, Logan, UT, USA) supplemented with 10% fetal bovine serum (FBS, Gibco, Grand Island, NY, USA) and 1% penicillin/streptomycin. HepG2 cells (low metastatic potential) were cultured in minimum essential medium (MEM, HyClone) supplemented with 10% FBS and 1% penicillin/streptomycin. HepG2-ISRE-Luc2 cells were established and maintained as described previously [20].

### 4.3. Luciferase Reporter Assay

HepG2-ISRE-Luc2 cells were seeded at 1 × 10^5^ cells/well in 96-well plates and incubated in a 5% CO_2_ incubator overnight. Before the addition of 1000 U/mL of IFN-α and subsequent incubation for 24 h, the cells were pretreated with the test compounds for 2 h. Thereafter, the cells were lysed in reporter lysis buffer, and luciferase activity was measured using the luciferase reporter assay system, according to the manufacturer’s instructions (Promega, Fitchburg, WI, USA). The luminescence intensity was measured using a Thermo Scientific Varioskan^®^ Flash (Thermo Fisher Scientific, Waltham, MA, USA).

### 4.4. Cell Viability Assay

Cells were seeded at 4 × 10^4^ cells/well in 96-well plates in 100 μL medium. Cultured cells were then treated with different concentrations of CIB-6 or a combination of CIB-6 and IFN-α. After 24 h, 10 μL of the Cell Counting Kit-8 (Beyotime, Shanghai, China) was added to the medium and incubated for another 2–4 h until the color changed from blue to pink. The relative fluorescence intensity in each well was measured using a Varioskan^®^ Flash (Thermo Fisher Scientific) at 450 nm.

### 4.5. Western Blotting

Cells were lysed using RIPA lysis buffer (Beyotime) containing phosphatase and protease inhibitor cocktail (Selleck) for 30 min. After centrifugation at 13,000× *g* for 5 min at 4 °C, the protein concentration was determined using a bicinchoninic acid (BCA) protein assay kit (Thermo Fisher Scientific, Waltham, MA, USA). Aliquots of total cell lysates were boiled with loading buffer and subjected to SDS-PAGE. Thereafter, proteins were blotted onto nitrocellulose membranes. The membranes were blocked with 5% BSA (Solarbio, Shanghai, China) at 25 °C for 1 h, then probed with each specific antibody (1:1000 dilution) overnight at 4 °C. An appropriate peroxidase-conjugated secondary antibody (1:2000 diluted) was then added for enhanced chemiluminescence detection (4A BIOTECH, Beijing, China).

### 4.6. Confocal Microscopy

SK-HEP-1 cells were seeded onto coverslips and treated with 25 μM CIB-6 or combined with 1000 U/mL IFN-α at 37 °C for 24 h. The cells were then fixed with 4% paraformaldehyde, permeabilized with 0.05% Triton X-100, and blocked with 5% BSA. Following incubation with anti-phospho-STAT3 (dilution 1:100) and Alexa Fluor 448 goat anti-rabbit IgG secondary antibody (Beyotime), the nuclei were stained with 4′,6-diamidino-2-phenylindole (DAPI, Beyotime) before mounting. Images were captured using a fluorescence microscope connected to a charge-coupled device camera (Leica DM6B, Wetzlar, Germany). The relative fluorescence intensities were quantified using ImageJ [56].

### 4.7. STAT3-Responsive Reporter Expression

Three STAT3 recognition repeat sequences (GTCGACATTTCCCGTAAATCGTCGA) were inserted into the pGL4.26 (Promega) to obtain pGL4.26-3×STAT3 plasmids (Xhol-3×STAT3-BglII primer: 5-tcgagGTCGACATTTCCCGTAAATCGTCGAGTCGACATTTCCCGTAAATCGTCGAGTCGACATTTCCCGTAAATCGTCGAa-3, BglII-3×STAT3-Xhol primer: 5-gatctTCGACGATTTACGGGAAATGTCGACTCGACGATTTACGGGAAATGTCGACTCGACGATTTACGGGAAATGTCGACc-3). Cells (2 × 10^5^ cells/well) were seeded into 24-well plates and transfected with 0.4 μg pGL4.26-3×STAT3 plasmid or pGL4.26 vehicle plasmid using TransIntro^TM^ EL Transfection Reagent (TransGen Biotech, Beijing, China). After 6 h of nucleofection, cells were treated with or without CIB-6. After 2 h, cells were treated with 1000 U/mL IFN-α for 24 h. The cells were then lysed in reporter lysis buffer and luciferase activity was measured using a luciferase reporter assay system according to the manufacturer’s instructions (Promega). The luminescence intensity was measured using a Thermo Scientific Varioskan^®^ Flash (Thermo Fisher Scientific).

### 4.8. Cell Thermal Shift Assay (CETSA)

Cultured cells were harvested and resuspended in phosphate buffer saline (PBS, HyClone) supplemented with 10% (*v*/*v*) protease inhibitor cocktail. The cell suspensions were then freeze-thawed three times in liquid nitrogen [57]. The lysates were extracted via centrifugation at 20,000× *g* for 20 min at 4 °C and divided into equal parts according to the concentration gradient of CIB-6 and the vehicle control. After 30 min of incubation at 37 °C, the respective lysates were divided into smaller (50 μL) aliquots, heated individually at different temperatures for 3 min, and cooled on ice for 3 min. The heated lysates were centrifuged at 20,000× *g* for 20 min at 4 °C to separate the soluble fractions from the precipitates. The supernatants were transferred to new microtubes and analyzed by SDS-PAGE.

### 4.9. Protein Expression and Purification

The cDNA of truncated STAT3 (Δ127-722) was cloned into the pET15b vector (Novagen, Darmstadt, Germany) with a 6×His-tag at the N-terminus (~70 kDa). The recombinant vector pET15b-STAT3 (Δ127-722) was transformed into BL21 (DE3) (TransGen Biotech) cells. Proteins were purified using nickel-chelating beads (CoWin Bioscience, Beijing, China) and eluted with 200 mM imidazole. The recombinant protein was then eluted from the washed resin using 10 mL of elution buffer (0.5 M imidazole, 20 mM Tris-HCl [pH 7.9], 0.5 M NaCl). All fusion proteins were dialyzed against 20 mM HEPES-NaOH buffer (pH 7.5) containing 150 mM NaCl and 1 mM tris-(2-carboxyethyl) phosphine (TCEP) overnight at 4 °C. Protein concentration was calculated using the BCA kit (Thermo Fisher Scientific).

### 4.10. Drug Affinity-Responsive Target Stability (DARTS)

The DARTS assay was performed according to a previously described protocol [58]. SK-HEP-1 cells were scraped and lysed in M-PER buffer (Pierce, Rockford, IL, USA) containing freshly added protease inhibitors for 10 min. After centrifugation at 18,000× *g* for 20 min at 4 °C, proteins were quantified via a BCA assay. The cell lysates or recombinant STAT3 protein (Δ127-722, 20 μg) was added to 10× TNC buffer (500 mM Tris-HCl (pH 8.0), 500 mM NaCl, 100 mM CaCl_2_) and equally divided between two tubes for 1 h at 25 °C with DMSO or CIB-6. After digestion with a certain proportion of pronase (Roche, Basal, Switzerland) at 25 °C for 30 min, digestion was terminated by adding protease inhibitors. Aliquots of samples were mixed with 5× loading buffer and boiled for SDS-PAGE.

### 4.11. Computer-Aided Virtual Calculation of the Molecular Properties

Molecular docking was carried out on the AutoDock software [59]. As a ligand, CIB-6 was subjected to a minimum energy calculation to determine its stable configuration. Human STAT3, a macromolecular receptor, was obtained from the Protein Data Bank (PDB ID: 6NJS). The entire protein was set as the calculation area to derive the free binding energy. The number of calculations was 100, and the result with the lowest free binding energy was selected for binding site analysis. The docking results were plotted using PyMOL software (grid center: −0.585, 53.79, 5.681).

### 4.12. Colony Formation Assay

HepG2, MHCC97L, and SK-HEP-1 cells in the logarithmic phase were seeded in 6-well plates at a density of 1000 cells/well. Cells were then treated with different concentrations of CIB-6 or 1000 U/mL IFN-α and allowed to proliferate for 7–14 days to form colonies. The cells were then fixed with methanol and stained with 0.1% crystal violet solution for 20 min. The colonies were recorded using a camera.

### 4.13. Wound Healing Assay

After HCCLM3 and SK-HEP-1 cells were seeded in 6-well plates at 5 × 10^5^ cells/well, a straight scratch was created using a 10-μL sterilized pipette tip. Thereafter, cell debris was washed with PBS. The cells were incubated in medium containing CIB-6 or 1000 U/mL IFN-α. Images were captured at the 0th hour and 24th hour using a Canon digital camera (×100).

### 4.14. Cell Migration and Matrigel Invasion Assays

HCCLM3 and SK-HEP-1 cells (1 × 10^5^) with 200 μL of serum-free culture medium were seeded onto the upper chamber (pre-coated with Matrigel (BD Biosciences, Franklin Lakes, NJ, USA) for cell invasion assays). Thereafter, 600 μL of DMEM (containing 10% FBS) was added to the lower chamber. After treatment with CIB-6, the cells could migrate or invade at 37 °C overnight. The upper chamber was swabbed with cotton, fixed with 4% methanol, and stained with 0.1% crystal violet. Graphic images were then captured with a microscope.

### 4.15. RNA Interference

SK-HEP-1 cells were seeded onto 6-well plates and transfected with siSTAT3 (siSTAT3 sense: 5-GAAUCAAGCAGUUUCUUCATT-3; siSTAT3 antisense: 5-UGAAGAAACUGCUUGAUUCTT-3, Tsingke Biotechnology, Beijing, China) by TransIntro^TM^ EL Transfection Reagent (TransGen Biotech) for 48 h. Thereafter, the cells were harvested for western blot and RT-qPCR.

### 4.16. Real-Time Quantitative PCR (RT-qPCR)

TRIzol reagent (Invitrogen, Carlsbad, CA, USA) was used to extract total cellular RNA according to the manufacturer’s instructions. RNA concentration was determined by examining the absorbance at 260 nm using a Varioskan Flash Multimode Reader (Thermo Fisher Scientific). Total RNA (1 μg) was reverse-transcribed using *TransScript^®^* Reverse Transcriptase with Oligo(dT)_18_ Primer (TransGen Biotech). Equal amounts of complementary DNA were subjected to real-time quantitative PCR with the florescent dye, SYBR Green I, according to the manufacturer’s protocol (TransGen Biotech). To eliminate differences in the number of cells, GAPDH was employed as the reference gene. Quantitative analyses were performed using the threshold cycle number (Ct), where the signal was detected above the background in the exponential phase. Relative RNA expression was analyzed by 2^–ΔΔC(t)^, and DMSO was used as a control. The sequences of the primer pairs (Tsingke Biotechnology) were: *STAT3*-F, 5-ACCAGCAGTATAGCCGCTTC-3; *STAT3*-R, 5-GCTTGGCGGATTAGCTCTTTT-3; *BTRC*-F, 5-TGCCCAAGCAACGGAAACT-3; *BTRC*-R, 5-GCCCATGTTGGTAATGACACA-3; *CTNNB1*-F, 5-CATCTACACAGTTTGATGCTGCT-3; *CTNNB1*-R, 5-GCAGTTTTGTCAGTTCAGGGA-3; *GAPDH*-F, 5-ACAACTTTGGTATCGTGGAAGG-3; *GAPDH*-R, 5-GCCATCACGCCACAGTTTC-3.

### 4.17. Animal Experiment

Five-week-old male BALB/c nude mice were purchased from Dashuo Laboratory Animal Technology Co., Ltd. (Chengdu, China). All animal experiments and procedures were performed in accordance with the Institutional Animal Care and Use Committee of Chengdu Institute of Biology, Chinese Academy of Sciences (CIBCAS-2018-016). Tumors were established via a subcutaneous injection of 5 × 10^6^ SK-HEP-1 cells into the flanks of mice. Thereafter, tumor volumes were estimated according to the formula: V= (L × W^2^)/2, where L is the longest and W is the shortest diameter of the tumor. When tumors reached 100 mm^3^, mice were randomly assigned to five groups, with each group containing eight mice. Mice in each group received a daily intraperitoneal (i.p.) injection of 200 μL vehicle control, 1 × 10^5^ U/kg IFN-α or different concentrations of CIB-6 (100 mg/kg with 75% olive oil, 15% normal saline solution, 5% ethanol, and 5% DMSO). The tumor dimensions and bodyweight of mice were documented every three days after the initial injection. After 24 days of treatment, the animals were sacrificed, and their organs and tumor specimens were removed.

### 4.18. Hematoxylin–Eosin (H&E) Staining and Immunohistochemistry

The tissues were fixed in 10% formalin solution, embedded in paraffin, and sectioned to 4 μm. The sections were then stained with H&E. For immunohistochemistry, antigen retrieval was carried out in 10 mM sodium citrate-hydrochloric acid buffer solution. Thereafter, endogenous peroxidase was blocked via a 15-min incubation in 3% H_2_O_2_ (methanol). Anti p-STAT3, anti-β-TrCP, and anti-β-catenin (1:50 dilution) were used for the immunohistochemical analysis. Tissues were incubated with the primary antibody at 4 °C overnight. After washing with PBS, the slices were incubated with the appropriate secondary antibody for 30 min at 37 °C. Peroxidase activity was revealed using 3,3-diaminobenzidine and counter-stained with hematoxylin. Images were captured using a BDS 200 microscope (CNOPTEC, Chongqing, China) and a camera (Canon, Tokyo, Japan).

### 4.19. Statistical Analysis

Statistical analyses were performed using the Student *t*-test, one-way, and two-way analyses of variance in GraphPad Prism 8.0. Reference [60] is presented as mean ± standard deviation (SD). Moreover, *p* < 0.05 was considered to indicate statistical significance.

## Supplementary Materials

The following are available online at https://www.mdpi.com/1422-0067/22/7/3354/s1.
