# Supplementary material for: A 2-Benzylmalonate Derivative as STAT3 Inhibitor Suppresses Tumor Growth in Hepatocellular Carcinoma by Upregulating β-TrCP E3 Ubiquitin Ligase
Source: Int J Mol Sci. 2021 Mar 25;22(7):3354. doi: 10.3390/ijms22073354 (PMC8036434; doi:10.3390/ijms22073354)

International Journal of
Molecular Sciences

MDPI

Article

A 2-benzylmalonate derivative as STAT3 inhibitor suppresses tumor growth in hepatocellular carcinoma by upregulating β-TrCP E3 ubiquitin ligase

Ting Peng [1,5], Orawan Wonganan [1], Zhonghui Zhang [2], Jialing Yu [1,5], Ruiying Xi [1,5], Yu Cao [1,5], Apichart Suksamrarn [3], Guolin Zhang [1,*] and Fei Wang [1,4,*]

[1] Center for Natural Products Research, Chengdu Institute of Biology, Chinese Academy of Sciences, Chengdu 610041, China; pengting@cib.ac.cn (T.P.); orawan.wog@mahidol.ac.th (O.W.); yujl@cib.ac.cn (J.Y.); xirui-ying18@mails.ucas.ac.cn (R.X.); caoyu@cib.ac.cn (Y.C.); zhanggl@cib.ac.cn (G.Z.); wangfei@cib.ac.cn (F.W);
[2] School of Chemistry Engineering, Sichuan University, Chengdu 610041, China; 2018223075220@stu.scu.edu.cn (Z.Z.);
[3] Department of Chemistry and Center of Excellence for Innovation in Chemistry, Faculty of Science, Ram-khamhaeng University, Bangkok 10240, Thailand; s_apichart@ru.ac.th (A.S.);
[4] Xiongan Institute of Innovation, Chinese Academy of Sciences, Hebei 071700, China;
[5] University of Chinese Academy of Sciences, Beijing 100049, China;
* Correspondence: wangfei@cib.ac.cn, Tel: +86 28 82890651 (F.W.); zhanggl@cib.ac.cn, Tel: +86 28 82890333 (G. Z.)

Support Information

Citation: Peng, T.; Wonganan, O.; Zhang, Z.; Yu, J.; Xi, R.; Cao, Y.; Suksamrarn, A.; Zhang, G.; Wang, F. A 2-Benzylmalonate Derivative as STAT3 Inhibitor Suppresses Tumor Growth in Hepatocellular Carcinoma by Upregulating β-TrCP E3 Ubiquitin Ligase. *Int. J. Mol. Sci.* **2021**, *22*, 3354. https://doi.org/10.3390/ijms22073354

Received: 19 February 2021
Accepted: 22 March 2021
Published: 25 March 2021

Publisher's Note: MDPI stays neutral with regard to jurisdictional claims in published maps and institutional affiliations.

Copyright: © 2021 by the authors. Submitted for possible open access publication under the terms and conditions of the Creative Commons Attribution (CC BY) license (http://creativecommons.org/licenses/by/4.0/).

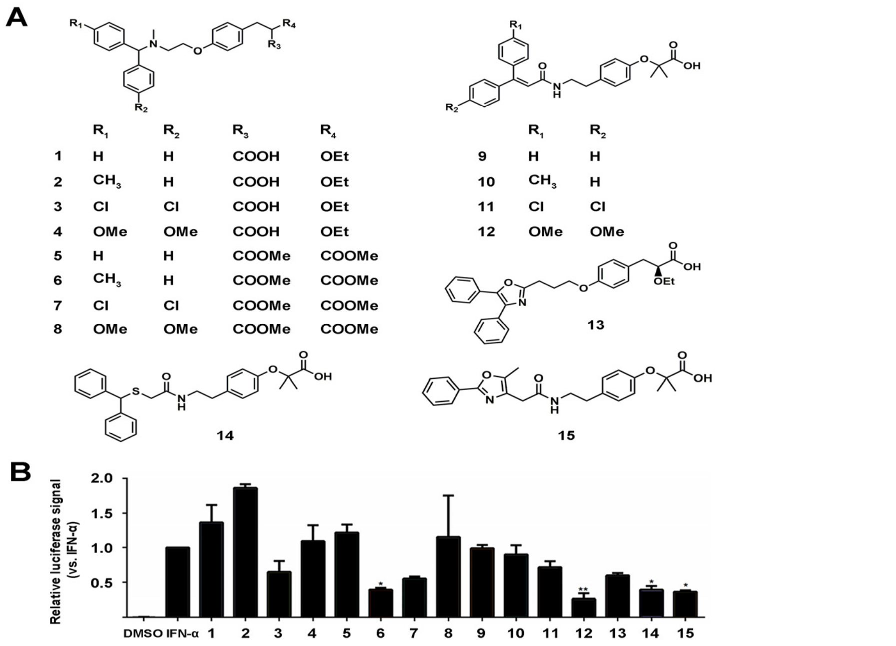

Figure S1. Identification of chemical compounds that modulate IFN-α signaling. **(A)** The chemical structures of the compounds. **(B)** HepG2-ISRE-luc2 cells were seeded in 96-well plates, treated with 10 μg/mL of compounds for 2 h, and administered 1000 U/mL IFN-α.

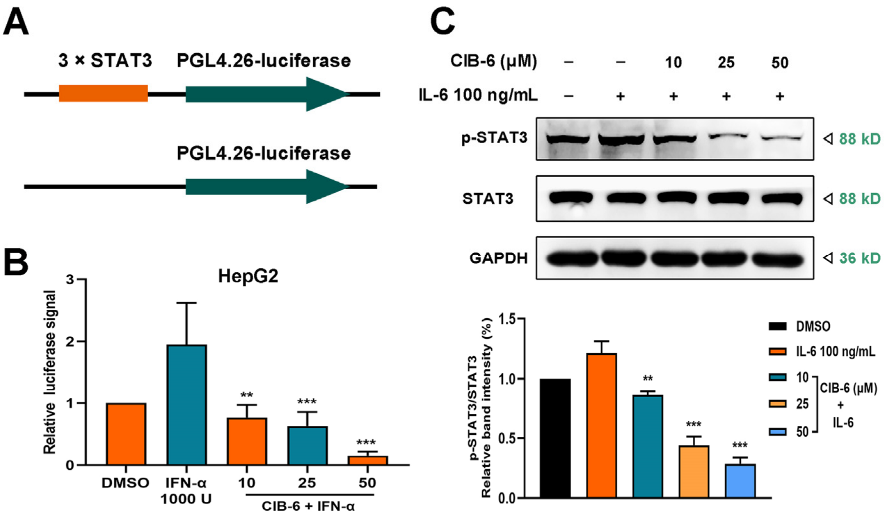

Figure S2. CIB-6 inhibits the activation of STAT3. (**A**) The scheme of pGL4.26-3×STAT3 plasmids. (**B**) HepG2 cells transfected pGL4.26-3×STAT3 plasmid, luciferase repression of CIB-6 treated cells compared with control or IFN-α was calculated and graphed as fold change in luciferase activity. Luciferase of empty vehicle was used to normalize the luciferase values. The data are presented as the mean ± SD of three independent experiments (** $p < 0.01$ and *** $p < 0.001$ as compared with IFN-α). (**C**) The lysates of SK-HEP-1 cellS treated with CIB-6 and 100 ng/mL IL-6 for 24 h were immunoblotted with p-STAT3 and total proteins of STAT3. GAPDH was used as an internal control. Bar graph shows quantified levels of p-STAT3/STAT3 from three independent experiments (** $p < 0.01$ and *** $p < 0.001$ as compared with IL-6).

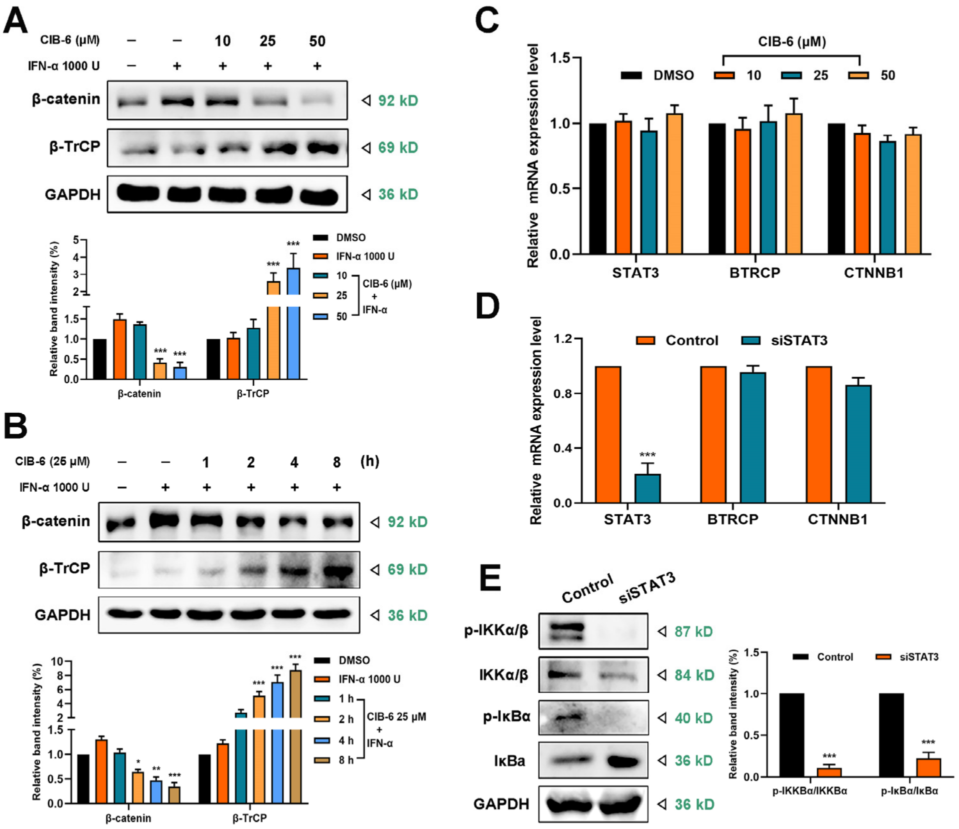

Figure S3. Effects of CIB-6 on β-catenin, β-TrCP and NF-κB pathway. (**A**) The SK-HEP-1 cells were treated with the indicated concentrations of CIB-6 for 24 h and 1000 U/mL IFN-α for 0.5 h. The cell lysates were immunoblotted with antibodies against β-catenin and β-TrCP. Bar graph shows quantified levels of proteins from three independent experiments (*** p < 0.001 as compared with IFN-α). (**B**) After the SK-HEP-1 cells were treated with 25 μM CIB-6 for the indicated period, immunoblots were generated to detect β-catenin and β-TrCP expression. Bar graph shows quantified levels of proteins from three independent experiments (* p < 0.05, ** p < 0.01, *** p < 0.001 as compared with IFN-α). (**C**) RT-qPCR detected the mRNA levels of *STAT3*, *BTRCP* and *CTNNB1* in SK-HEP-1 cell after treated with indicated concentrations of CIB-6 for 24 h. *GAPDH* were used for normalization. SK-HEP-1 cells transiently transfected with siSTAT3. (**D**) The mRNA level of *STAT3*, *CTNNB1* and *NFKB1* were examined using RT-qPCR. The data are presented as the mean ± SD of three independent experiments (*** p < 0.001 as compared with control). (**E**) The cell lysates were immunoblotted with antibodies against phosphorylated and total proteins of IKKα/β and IκBα. Bar graph shows quantified levels of p-IKKα/β / IKKα/β and p-IκBα / IκBα from three independent experiments (*** p < 0.001 as compared with control).

Uncropped Western Blots images

Figure 1D

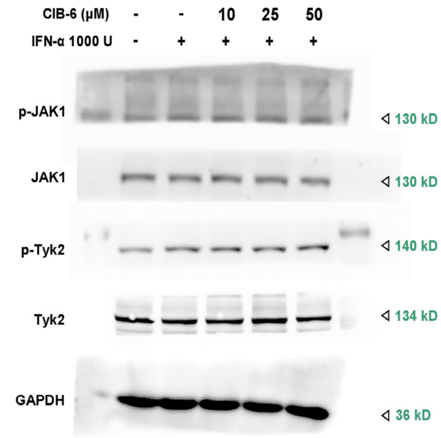

Figure 1F

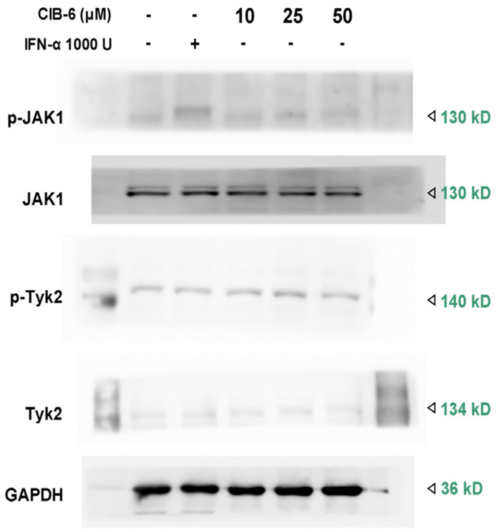

Figure 1E

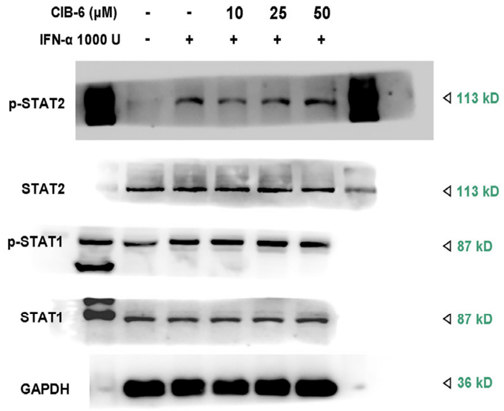

Figure 2A

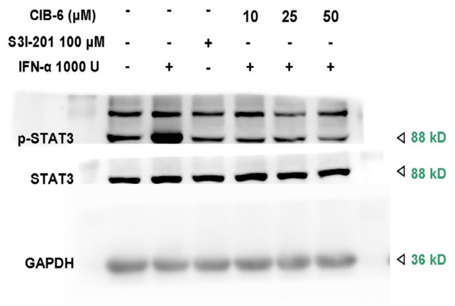

Figure 2B

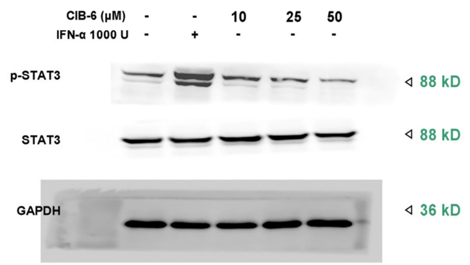

Figure 2E

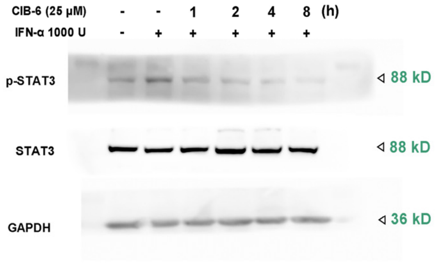

Figure 4H

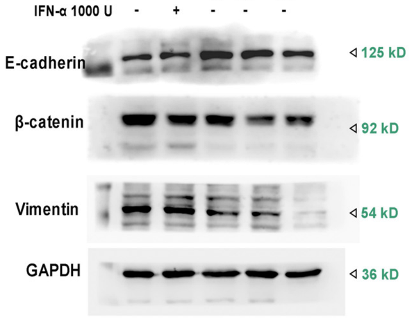

Figure 3A

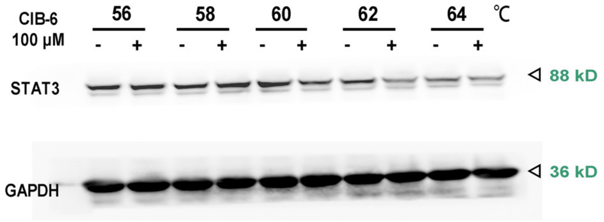

Figure 3B

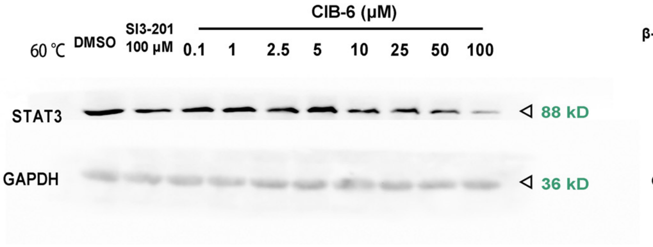

Figure 5A

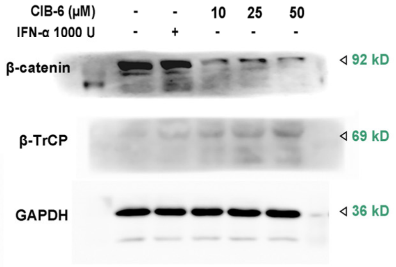

Figure 5B

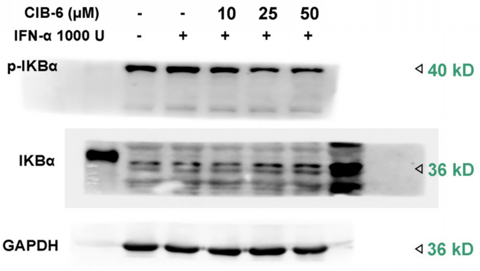

Figure 5C

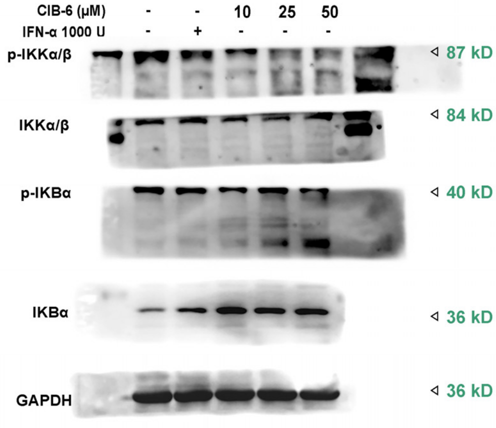

Figure 5D

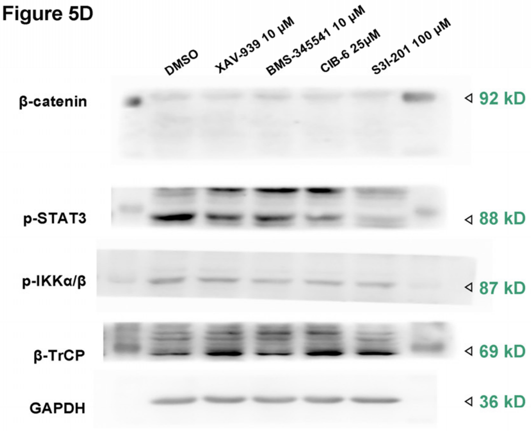

Figure 5E

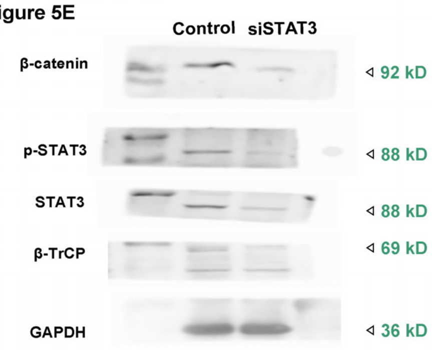

Figure S2C

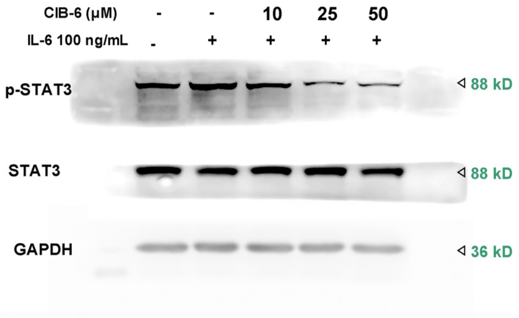

Figure S3B

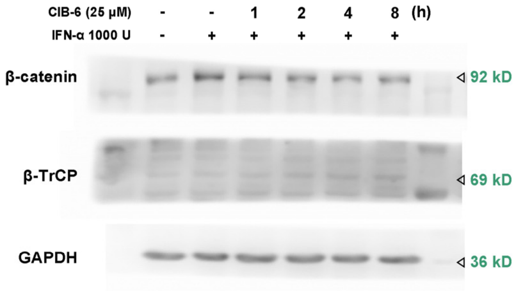

Figure S3A

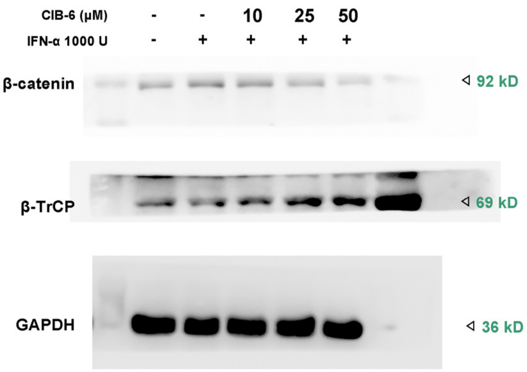

Figure S3E

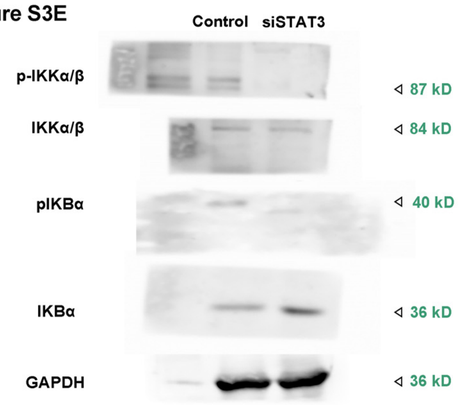

Supplement: Supplementary file 1 [file ijms-22-03354-s001.pdf]
